# Supplementary figures and images for: Binge alcohol drinking before pregnancy is closely associated with the development of macrosomia: Korean pregnancy registry cohort
Source: PLoS One. 2022 Jul 12;17(7):e0271291. doi: 10.1371/journal.pone.0271291 (PMC9275693; doi:10.1371/journal.pone.0271291)

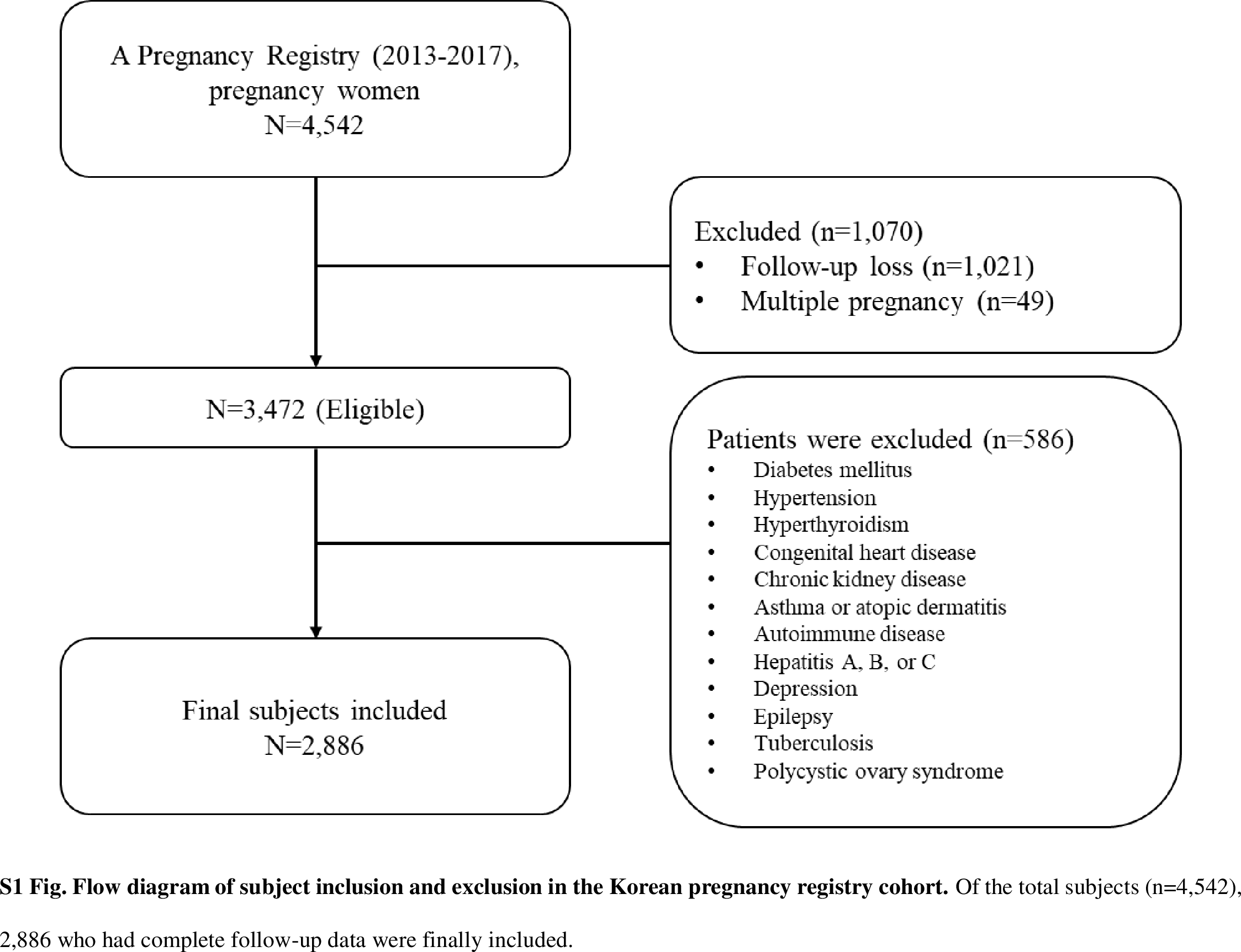

Supplement: S1 Fig — Of the total subjects (n = 4,542), 2,886 who had complete follow-up data were finally included. (TIF) [file pone.0271291.s001.tif]

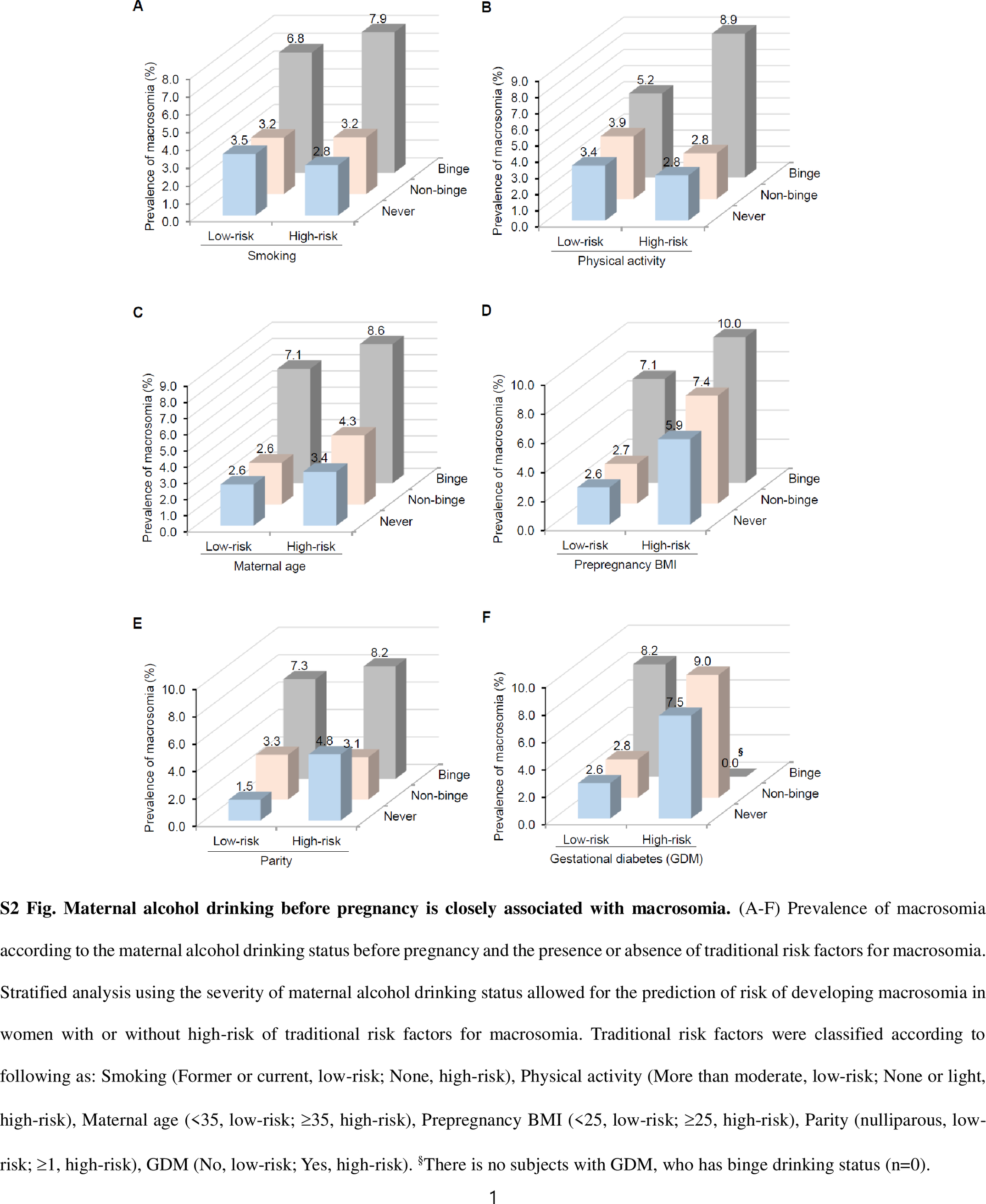

Supplement: S2 Fig — (A-F) Prevalence of macrosomia according to the maternal alcohol drinking status before pregnancy and the presence or absence of traditional risk factors for macrosomia. Stratified analysis using the severity of maternal alcohol drinking status allowed for the prediction of risk of developing macrosomia in women with or without high-risk of traditional risk factors for macrosomia. Traditional risk factors were classified according to following as: Smoking (Former or current, low-risk; None, high-risk), Physical activity (More than moderate, low-risk; None or light, high-risk), Maternal age (<35, low-risk; ≥35, high-risk), Prepregnancy BMI (<25, low-risk; ≥25, high-risk), Parity (nulliparous, low-risk; ≥1, high-risk), GDM (No, low-risk; Yes, high-risk). §There is no subjects with GDM, who has binge drinking status (n = 0). (TIF) [file pone.0271291.s002.tif]

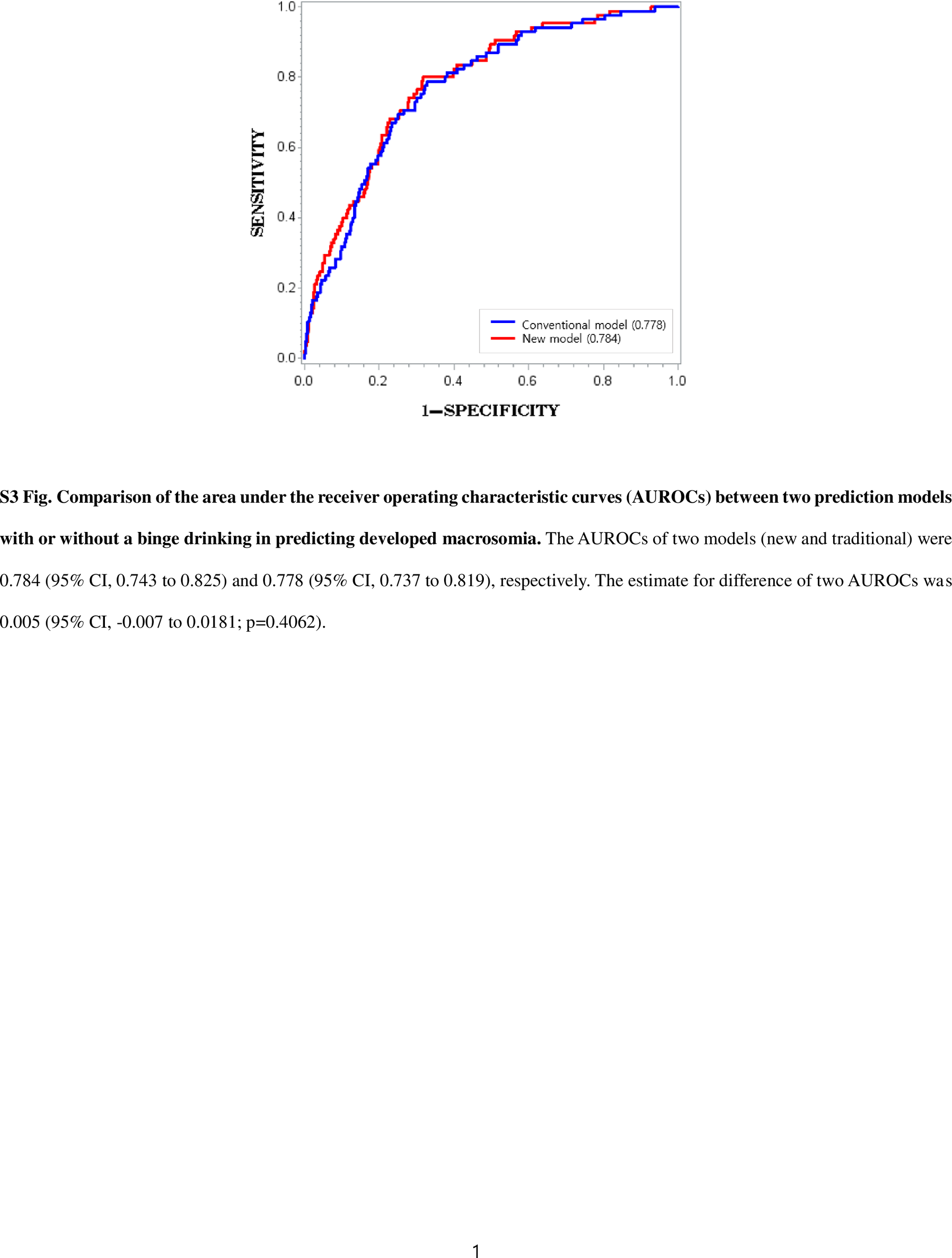

Supplement: S3 Fig — The AUROCs of two models (new and traditional) were 0.784 (95% CI, 0.743 to 0.825) and 0.778 (95% CI, 0.737 to 0.819), respectively. The estimate for difference of two AUROCs was 0.005 (95% CI, -0.007 to 0.0181; p = 0.4062). (TIF) [file pone.0271291.s003.tif]
