# Supplementary material for: Binge alcohol drinking before pregnancy is closely associated with the development of macrosomia: Korean pregnancy registry cohort
Source: PLoS One. 2022 Jul 12;17(7):e0271291. doi: 10.1371/journal.pone.0271291 (PMC9275693; doi:10.1371/journal.pone.0271291)
Supplement: S1 Table — (DOCX) [file pone.0271291.s004.docx]

**S1 Table. Offspring’s characteristics and outcomes according to maternal alcohol-drinking status before pregnancy in 2,554 participants excluding 332 women who drank alcohol in the first trimester (related to Table 4).**

|  |  | All participants (n=2,554) |  | Never drinking (n=561) | Ever drinker^†^ | | *p*-value |
| --- | --- | --- | --- | --- | --- | --- | --- |
|  |  |  |  |  | Non-binge drinking (n=1,776) | Binge drinking (n=217) |  |
| Gender | | | | | | | |
| Boys |  | 1308(51.2) |  | 293 (52.2) | 899(50.6) | 116(53.5) | 0.561 |
| Girls |  | 1246(48.8) |  | 268 (47.8) | 877(49.4) | 101(46.5) |  |
| Weight (g) |  | 3238.3 ± 412.4 |  | 3224.6 ± 441.6^a^ | 3235.2 ± 514.2^a^ | 3323.4 ± 387.3^b^ | **0.012** |
| Height (cm) |  | 49.5 ± 3.4 |  | 49.5 ± 2.2 | 49.5 ± 1.6 | 49.7 ± 2.1 | 0.427 |
| Head circumference (cm) |  | 34.4 ± 2.8 |  | 34.4 ± 1.4 | 34.5 ± 1.2 | 34.4 ± 3.5 | 0.224 |
| Glucose (mg/dl)^‡^ |  | 82.8 ± 16.4 |  | 80.5 ± 16.9 | 83.4 ± 22.4 | 81.5 ± 14.2 | 0.098 |
| Macrosomia | | | | | | | |
| No |  | 2457 (96.2) |  | 545 (97.1) | 1711 (96.3) | 201 (92.6) | **0.002** |
| Yes |  | 97(3.8) |  | 16 (2.9) | 65 (3.7) | 16 (7.4) |  |
| Congenital anomaly | |  |  |  |  |  |  |
| No |  | 2504 (98.0) |  | 550 (98.0) | 1739 (97.9) | 215 (99.1) | 0.634^§^ |
| Yes |  | 50 (2.0) |  | 11 (2.0) | 37 (2.1) | 2 (0.9) |  |
| Admissions to the intensive care unit | | | | | | | |
| No |  | 2241 (87.7) |  | 485 (86.5) | 1571 (88.5) | 185 (85.3) | **0.011** |
| Yes |  | 313 (12.3) |  | 76 (13.5) | 205 (11.5) | 32 (14.7) |  |
| Apgar score | | | | | | | |
| 1 minute, mean |  | 7.95 ± 0.6 |  | 7.93 ± 0.7 | 7.96 ± 0.5 | 7.90 ± 0.9 | 0.357 |
| 5 minute, mean |  | 8.81 ± 0.5 |  | 8.76 ± 0.6 | 8.81 ± 0.7 | 8.73 ± 0.6 | 0.234 |

We re-analyzed offspring’s characteristics and outcomes for 2,554 participants excluding 332 women who drank alcohol in the first trimester based on Table 4 (n=2,886). Data are expressed as mean ± standard deviation (SD) or n (%). The *p*-value is a comparison between the three groups. Bold values are statistically significant findings (p<0.05).

^a,b^Different letters represent statistical difference by Tukey’s multiple comparison test.

^†^Ever drinker included 1,993 participants (Non-binge drinking 1,776 and Binge drinking 217). The 332 women who drank alcohol in the first trimester are excluded from non-binge drinking groups (n=323) and binge drinking groups (n-9).

^‡^Only 1,039 offspring were included in the analysis. ^§^The *p*-value is calculated by Fisher's exact test.
