# Supplementary material for: Binge alcohol drinking before pregnancy is closely associated with the development of macrosomia: Korean pregnancy registry cohort
Source: PLoS One. 2022 Jul 12;17(7):e0271291. doi: 10.1371/journal.pone.0271291 (PMC9275693; doi:10.1371/journal.pone.0271291)
Supplement: S2 Table — (DOCX) [file pone.0271291.s005.docx]

**S2 Table. Odds ratio with 95% CIs of macrosomia depending on maternal alcohol-drinking status before pregnancy in 2,554 participants excluding 332 women who drank alcohol in the first trimester (related to Table 5).**

|  |  | *No. of subjects* |  | Never drinking  (n=561) | Ever drinker (n=1,993)† | | | |
| --- | --- | --- | --- | --- | --- | --- | --- | --- |
|  |  |  |  |  | Non-binge drinking  (n=1,776) | | Binge drinking  (n=217) | |
|  |  |  |  |  | OR (95% CI) | *p* value | OR (95% CI) | *p* value |
| Macrosomia (>4,000g) | | | | |  |  |  |  |
| Unadjusted |  | *2,554* |  | 1.00 | 1.15 (0.68-1.99) | 0.627 | 2.78 (1.35-5.72) | 0.003 |
| Model 1 |  | *2,554* |  | 1.00 | 1.17 (0.67-1.98) | 0.616 | 2.89 (1.41-5.86) | 0.003 |
| Model 2 |  | *2,554* |  | 1.00 | 1.10 (0.66-1.85) | 0.744 | 2.85 (1.36-5.87) | 0.005 |
| Model 3 |  | *2,554* |  | 1.00 | 1.07 (0.62-1.83) | 0.857 | 2.28 (1.06-4.85) | 0.032 |

We re-assessed the ORs depending on alcohol-drinking status for offspring macrosomia using multivariable logistic regression analyses for 2,554 participants excluding 332 women who drank alcohol in the first trimester based on Table 5 (n=2,886). Data are OR (95% CI) for unadjusted and adjusted models 1-3.

Model 1 adjusted for maternal age, education and monthly income

Model 2 adjusted for maternal age, education, monthly income, smoking and physical activity

Model 3 adjusted for maternal age, education, monthly income, smoking and physical activity, gestational age, pre-pregnancy body mass index, parity, offspring's gender and gestational diabetes

^†^ Ever drinker included former (n=1,993) and current drinker (n=0). OR, odds ratios; CI, confidence interval.
