# Supplementary material for: Binge alcohol drinking before pregnancy is closely associated with the development of macrosomia: Korean pregnancy registry cohort
Source: PLoS One. 2022 Jul 12;17(7):e0271291. doi: 10.1371/journal.pone.0271291 (PMC9275693; doi:10.1371/journal.pone.0271291)
Supplement: S3 Table — (DOCX) [file pone.0271291.s006.docx]

**S3 Table. Odds ratio with 95% CIs of macrosomia depending on maternal alcohol-drinking status before pregnancy in 2,746 participants who had valid data for all potential confounders (related to Table 5).**

|  |  | *No. of subjects* |  | Never drinking  (n=523) | Ever drinker (n=2,223)† | | | |
| --- | --- | --- | --- | --- | --- | --- | --- | --- |
|  |  |  |  |  | Non-binge drinking (n=1,998) | | Binge drinking (n=225) | |
|  |  |  |  |  | OR (95% CI) | *p* value | OR (95% CI) | *p* value |
| Macrosomia (>4,000g) | | | | |  |  |  |  |
| Unadjusted |  | *2,746* |  | 1.00 | 1.13 (0.68-1.94) | 0.681 | 2.78 (1.36-5.61) | 0.004 |
| Model 1 |  | *2,746* |  | 1.00 | 1.15 (0.70-1.96) | 0.667 | 2.89 (1.43-5.87) | 0.003 |
| Model 2 |  | *2,746* |  | 1.00 | 1.07 (0.64-1.87) | 0.788 | 2.87 (1.40-5.91 | 0.005 |
| Model 3 |  | *2,746* |  | 1.00 | 1.03 (0.58-1.81) | 0.967 | 2.31 (1.09-4.88) | 0.029 |

We re-assessed the ORs depending on alcohol-drinking status for offspring macrosomia using multivariable logistic regression analyses for 2,746 participants who had valid data for all confounders based on Table 5 (n=2,886). Data are OR (95% CI) for unadjusted and adjusted models 1-3.

Model 1 adjusted for maternal age, education and monthly income

Model 2 adjusted for maternal age, education, monthly income, smoking and physical activity

Model 3 adjusted for maternal age, education, monthly income, smoking and physical activity, gestational age, pre-pregnancy body mass index, parity, offspring's gender and gestational diabetes

^†^ Ever drinker included former (n=2,222) and current drinker (n=1). OR, odds ratios; CI, confidence interval.
