# Supplementary material for: Binge alcohol drinking before pregnancy is closely associated with the development of macrosomia: Korean pregnancy registry cohort
Source: PLoS One. 2022 Jul 12;17(7):e0271291. doi: 10.1371/journal.pone.0271291 (PMC9275693; doi:10.1371/journal.pone.0271291)
Supplement: S4 Table — (DOCX) [file pone.0271291.s007.docx]

**S4 Table. Multivariable-adjusted ORs of developing macrosomia for the risk factors of macrosomia.**

|  | Unadjusted | *p*-value | Adjusted^†^ | *p*-value |
| --- | --- | --- | --- | --- |
|  | OR (95% CI) |  | OR (95% CI) |  |
| **Alcohol-drinking status** |  |  |  |  |
| Never drinking | 1.00 |  | 1.00 |  |
| Non-binge drinking | 1.12 (0.65-1.95) | 0.681 | 1.01 (0.57-1.80) | 0.968 |
| Binge drinking | 2.77 (1.37-5.59) | **0.004** | 2.29 (1.08-4.86) | **0.031** |
| **Maternal age (year)** | 1.06 (1.01-1.12) | **0.030** | 1.05 (0.99-1.11) | 0.121 |
| **Prepregnancy BMI (kg/m^2^)** | 1.15 (1.09-1.21) | **<.0001** | 1.14 (1.07-1.21) | **<.0001** |
| **Parity (the number of deliveries)** | 1.23 (0.91-1.67) | 0.179 | 1.35 (0.97-1.87) | 0.074 |
| **Gestational age (weeks)** | 1.73 (1.44-2.09) | **<.0001** | 1.92 (1.57-2.35) | **<.0001** |
| **Newborn's gender** |  |  |  |  |
| Male | 1.80 (1.19-2.74) | **0.006** | 2.04 (1.32-3.17) | **0.001** |
| Female | 1.00 |  | 1.00 |  |
| **Education** |  |  |  |  |
| High school or less | 1.00 |  | 1.00 |  |
| College | 1.22 (0.56-2.68) | 0.617 | 1.63 (0.70-3.81) | 0.260 |
| Graduate school or more | 1.11 (0.45-2.73) | 0.822 | 1.56 (0.59-4.12) | 0.374 |
| **Monthly income (KRW)** |  |  |  |  |
| Low (<3 million) | 1.00 |  | 1.00 |  |
| Mid-low (3-4 million) | 1.22 (0.59-2.53) | 0.590 | 1.39 (0.64-3.01) | 0.410 |
| Mid-high (4-5 million) | 1.02 (0.49-2.09) | 0.969 | 1.33 (0.62-2.84) | 0.468 |
| High (>5 million) | 1.01 (0.53-1.92) | 0.986 | 1.15 (0.57-2.31) | 0.692 |
| **Smoking** |  |  |  |  |
| None | 1.00 |  | 1.00 |  |
| Former or current | 1.22 (0.66-2.26) | 0.526 | 1.00 (0.52-1.95) | 0.998 |
| **Physical activity** |  |  |  |  |
| None or light | 1.00 |  | 1.00 |  |
| More than moderate | 0.86 (0.57-1.29) | 0.462 | 0.88 (0.57-1.35) | 0.557 |
| **Gestational diabetes** |  |  |  |  |
| No | 1.00 |  | 1.00 |  |
| Yes | 2.53 (1.43-4.47) | **0.001** | 1.90 (1.02-3.55) | **0.043** |

Bold values are statistically significant findings (p<0.05).

BMI, body mass index; OR (95% CI), Odds ratio (95% confidence interval). Bold values are statistically significant findings (p<0.05).

^†^Adjusted for multiple variables each other in the table. OR, odds ratio; CI, confidence interval. BMI, body mass index. KRW, Korean Won
