# Supplementary material for: Binge alcohol drinking before pregnancy is closely associated with the development of macrosomia: Korean pregnancy registry cohort
Source: PLoS One. 2022 Jul 12;17(7):e0271291. doi: 10.1371/journal.pone.0271291 (PMC9275693; doi:10.1371/journal.pone.0271291)
Supplement: Attachment — Submitted filename: Response to Reviewers.docx [file pone.0271291.s008.docx]

May 13, 2022

Editor Board

*PLOS ONE*

Dear Editors,

I would like to re-submit a revised version of the manuscript for publication in *PLOS One*, titled ‘Binge alcohol drinking before pregnancy is closely associated with the development of macrosomia: Korean pregnancy registry cohort’. The manuscript ID is PONE-D-21-31508R1.

We appreciate you giving us the opportunity to revise our manuscript. We have carefully considered each of the editor’s and reviewers’ comments and suggestions, which helped us improve our manuscript. We have provided point-by-point responses to all the reviewers’ comments in the attached letter.

We hope that our revised manuscript is now suitable for publication in *PLOS One*. We look forward to hearing from you.

Sincerely yours,

Won-Ho Kim, Ph.D.

Director

Division of Cardiovascular Disease Research,

Department of Chronic Disease Convergence Research, National Institute of Health,

187, Osong Saengmyeong 2-ro, Osong-eup, Cheongju city,

Chungbuk, Korea, 28159,

Tel: +82-43-719-8650; Fax: +82-43-719-8689; E-mail: [jhkwh@nih.go.kr](mailto:jhkwh@nih.go.kr)

**Responses to the Editor’s and Reviewers’ Comments**

I. **EDITOR’S COMMENTS TO AUTHOR:**
1. While the reviewers overall feel the paper has merit, they also provided a large number of specific suggestions that the authors should address. In addition, we recommend additional English-language editing.

**→ Response:** According to your suggestion, we have carefully reviewed and reorganized the manuscript. We have also provided point-by-point responses to the reviewers’ comments in the attached letter. As well, English-language editing was performed by America Journal of Expert.

2. Please include the following items when submitting your revised manuscript:
   2.1. A rebuttal letter that responds to each point raised by the academic editor and reviewer(s). You should upload this letter as a separate file labeled 'Response to Reviewers'.

**→ Yes, we have uploaded with a separate file labeled “Response to Reviewers”.**

2.2. A marked-up copy of your manuscript that highlights changes made to the original version. You should upload this as a separate file labeled 'Revised Manuscript with Track Changes'.

**→ Yes, we have uploaded with a separate file labeled “Revised Manuscript with Track Changes”.**

2.2. An unmarked version of your revised paper without tracked changes. You should upload this as a separate file labeled 'Manuscript'.

**→ Yes, we have uploaded with a separate file labeled “Manuscript”.**

3. **Journal Requirements**
   3.1. Please ensure that your manuscript meets PLOS ONE's style requirements, including those for file naming.

**→ Response:** The manuscript was changed and corrected according to PLOS ONE's style requirements.

3.2. Please provide additional details regarding participant consent. In the ethics statement in the Methods and online submission information, please ensure that you have specified whether consent was written or verbal/oral.

**→ Response:** According to your suggestion, we have revised and changed the section of **Study approval in Methods**. The section was changed into “All Participants provided written informed consent, and the study protocol was approved by the Institutional Review Board (IRB) of Cheil General Hospital (IRB number: CGH-IRB-2013-10), CHA Gangnam, Medical Center (IRB number: 2013-14-KNC12-018), and the Korea Centers for Disease Control and Prevention (IRB No. 2013-10EXP-02-P-E) separately. It was emphasized to all participants to all participants that they were free to withdraw from any part of the study at any point in time.”**(Page 9-10, line 183-188)**.

3.3. In your Data Availability statement, you have not specified where the minimal data set underlying the results described in your manuscript can be found.

**→ Response:** We added our **data availability** statement in **Page 28, line 524-531** as follows: “The datasets analyzed during the current study are not publicly available due to current Korean law regulating release of personal information, even in the cases of research purposes. All data are currently only available to the researchers participated in the establishment of Korean pregnancy registry study; however, data analysis collaborations may be possible through specific research proposals. And also, the datasets used and analyzed during the current study are available from the corresponding author on reasonable request and requested data may be disclosed only after approval from the Institutional Review Board (IRB) of Cheil General Hospital and CHA Gangnam, Medical Center, Seoul, Korea. Further information can be requested by e-mailing the principal investigator (hmryu2012@naver.com).”

**II. Reviewer #1 :**

**<In Method>**

1. I recommend giving more information about the study population. For example, how and where (hospital?) were pregnant women recruited into the registry? The registry cohort seems to target all pregnant women in Korea, but it only recruited 4542 pregnant women between 2013-2017. This is important because it determines whether the study sample is a representative sample of the general population.

**→ Response:** According to your suggestion, we described detailed information about how and where were pregnant women recruited into the registry as follows: “Korean Pregnancy Registry Cohort was established in 2013 to investigate the prevalence and risk factors of pregnancy complications among Korean pregnant women and it is the only one established with support from Korea National Institute of Health, Korea Disease Control and Prevention Agency (KNIH-KDCA). Between March 2013 to January 2017, all pregnant women who visited Cheil General Hospital and CHA Hospital for antenatal care during the first trimester were asked to participate in the study. These two hospitals are representative obstetrics and gynecology hospitals located in Seoul, the capital of South Korea, and approximately 5,000 and 2,500 deliveries take place at these facilities per year, respectively. Trained research nurses explained the study in detail and obtained written informed consent from participants and assisted them in completing the interview-based questionnaires. The approved pregnant women were enrolled at first trimester (around 8-13 weeks’ gestation) and re-visited during the following their gestation period at second trimester (around 24-28 weeks’ gestation), third trimester (around 36-40 weeks’ gestation), delivery, and postpartum, respectively.” **(Page 6, line 93-102)**

**----** Contrary to your comments, the Pregnancy Registry Cohort used in this study did not register to register all pregnant women in Korea. In addition, it cannot be said to represent all pregnant women in Korea, since the registration of pregnant women in this cohort was carried out only at two hospitals, Cheil General Hospital and CHA Hospital. However, these hospitals are representative obstetrics and gynecology hospitals located in Seoul and approximately 5,000 and 2,500 deliveries take place at these facilities per year, respectively. These hospitals have the highest number of antenatal care visits for pregnancy and childbirth in Korea. In addition, this registry cohort is the only national pregnancy registry cohort established with the support of the Korea Government (Korea National Institute of Health, Korea Disease Control and Prevention Agency (KNIH-KDCA) and long-term follow-up studies are ongoing. So, this cohort will be a representative cohort that will produce many outcomes related to pregnancy and childbirth in Korea and thereafter, to offspring’s growth and development.

2. “Of those, we initially selected 3,472 pregnancies with singleton and complete follow-up data (exclude those with follow-up loss (n=1,021) and multiple pregnancy (n=49))”. Were the 3,472 pregnancies those who had valid (non-missing) data on both binge drink and macrosomia? The inclusion criteria in this step should be provided in the text.

**→ Response:** As suggested, the inclusion criteria for the selection of participants were described in the text of Methods as follows: “Of those, we initially selected 3,472 pregnancies with singleton and complete follow-up data (include those with valid (non-missing) data on both binge drinking and macrosomia and exclude those with follow-up loss (n=1,021) and multiple pregnancy (n=49)).” **(Page 6, line 103-105)**

3. I recommend providing the reasons for excluding participants with pre-existing disease before pregnancy.

**→ Response:** Exclusion criteria often include subjects who have a threshold severity of disease and have certain other conditions, or who are using medications or factors that could mask the effect of the intervention or certain factors. Therefore, to minimize heterogeneity (“noise”), which can mask the effect of the intervention or certain factors, our study excluded patients with pre-existing disease before pregnancy from total participants. **(Page 6, line 106)**

4. In the following sentence: “If women answered ‘Former drinker’ or ‘Current drinker’, they were then further asked average frequency of alcohol-drinking (≤1/month, 1-2/month, 2-3/week, ≥4/week, everyday) and quantity of alcohol-drinking per drinking day (1-2 drinks, 3-4 drinks, 5-6 drinks, 7-9 drinks, ≥10 drinks).” Were pregnant women asked about their average frequency of alcohol consumption in their lifetime? in the past year? 6 months? Or 1 month?

**→ Response:** In cohort, we collected the information on maternal alcohol consumption before and during pregnancy through a questionnaire for lifetime drinking, past year, 6 months, recent 1month, current drinking, duration, and amount at the first antenatal care visit and also continuously evaluated at each visit. In this study, the average frequency of alcohol drinking and quantity of alcohol-drinking per drinking day were evaluated based on the result for a specific questionnaire in their lifetime before pregnancy. In fact, there was few participant to drink alcohol during the pregnancy (n=3) in this cohort. **(Described in page 7, line 117)**

5. In the “definition of macrosomia and its risk factors” section, I recommend specifying whether “smoking” and “physical activity” refer to smoking and physical activity before pregnancy or during pregnancy. Provide definitions of different levels of physical activity.

**→ Response:** As like in evaluation of alcohol drinking, smoking and physical activity were also assessed at each visit. Smoking was queried by the following items such as lifetime, last month, and current smoking and their duration and amount. And also, physical activity was queried at each visit for physical activity before and during pregnancy (frequency and duration of walking, moderate and vigorous-intensity activity). So, the results for smoking or physical activity in their lifetimes including before and during pregnancy were used for assessing infant outcome, macrosomia.

Since smoking and physical activity are not included in traditional risk factors of macorosomia, we described in **page 8, line 148-151** as follows: “In addition, modifiable lifestyle factors were classified according to following as: Smoking before and during pregnancy (Former or current, low-risk; None, high-risk), Physical activity before and during pregnancy (More than moderate, low-risk; None or light, high-risk); and analyzed their effects on the risk of developing macrosomia by maternal alcohol drinking status.”.

**<Results>**

1. Include the association results between binge drinking and macrosomia, excluding 332 women who drank during the first trimester in the supplementary file.

**→ Response:** 332 women stopped drinking within the first trimester when they found out that they are pregnant, only 3 women drank alcohol at the time of registration in the first trimester. As you suggested, in other to more accurately confirm the relationship between pre-pregnancy drinking and macrosomia, we reanalyzed these relationships for 2,554 women excluding 332 women who drank alcohol in the first trimester of pregnancy. The results were very similar to the results of an analysis of 2,886 subjects including them. As shown in Table S1, there was little difference in the prevalence of macrosomia, and there was no significant change in other characteristics.

These are described in **Page 15, line 262-265** as follows: “Meanwhile, to confirm the effect of pre-pregnancy drinking on the offspring’s characteristics and outcomes, 2,554 participants excluding 332 women who drank alcohol in the first trimester were analyzed. As shown in S1 Table, compared with the results analyzed in 2,886 participants (Table 4), there was little difference in the prevalence of macrosomia and other outcomes.”

On the other hand, we also reanalyzed the relative risk of macrosomia predicted by maternal alcohol-drinking status in 2,554 participants excluded 332 women who drank during the first trimester. The unadjusted and adjusted odds ratio (OR) for developing macrosomia was also similar to the results of 2,886 women including women who drank alcohol in the first trimester. The results are shown in S2 Table. These are described in **Page 17, line 302-303** as follows: “Also, similar results were obtained in analysis for 2,554 participants excluding 332 women who drank alcohol during the first trimester of pregnancy (S2 Table)”

2. Table 2 shows the lab results from 1st and 3rd trimesters.

2.1. Recommend specifying whether glucose results reflect fasting or non-fasting glucose level.

**→ Response:** As you pointed, glucose is fasting blood glucose. We have corrected and changed from Glucose into FBG in Table 2 and described in table footnotes as follows: FBG, fasting blood glucose **(Page 13, table 2, line 229)**

2.2. recommend specifying whether the p-values reflect the comparison between the never drinking and ever drinker group or the comparison across the three groups.

**→ Response:** We described in the footnotes of Table 2 **(Page 13, line 224)** as follows: “The *p*-value is a comparison between the three groups”. **And also, the description was added in the footnotes of Table 1-4 and S1 Table.**

3. In table 3, the authors provided the results of the association between alcohol intake and obstetric outcome. Definitions of obstetric outcomes, such as pregnancy-induced hypertension and perinatal/postpartum depression, should be provided.

→ Response: Definition for pregnancy-induced hypertension and perinatal/postpartum depression was described in the footnotes of table 3. (**Page 14, Table 3, line 246-249**)

^‡^Pregnancy-induced hypertension was defined by a systolic blood pressure ≥140 mmHg and/or diastolic blood pressure ≥90 mmHg without proteinuria (<0.3 g in a 24-hour urine collection) and the hypertension must have developmed after 20 weeks of gestation.

^§^Perinatal/postpartum depression were defined by a score of ≥10 on K-EPDS (Modified Korean-Edinburgh Postnatal Depression Scale) during pregnancy or in the 4 weeks following delivery, respectively.

4. In table 5, the same group of participants should be used when comparing different models to make the results comparable. For example, I suggest the authors provide results of the unadjusted model for all 2886 participants and 2746 participants who had valid data for all potential confounders, respectively. Provide results of models 1-3 for the 2746 participants.

→ Response: As you suggested, we have additively analyzed the relative risk of significant macrosomia for 2,746 participants who had valid data for all potential confounders and their results were added and shown in **S3 Table (Supplementary Information Page 7)**. 2746 participants who had valid data for all potential confounders are subdivided to Never drinking (n=523), Non-binge drinking (n=1,998), and binge drinking (n=225), respectively. As shown in S3 Table, the relative risk of macrosomia using model 1, 2, 3 are very similar with those for 2,886 participants (Table 5).

Described in **page 17, line 302-304** as follows: “Also, similar results were obtained in analysis for 2,554 participants excluding 332 women who drank alcohol during the first trimester of pregnancy (S2 Table) and 2,746 participants who had valid data for all potential confounders (S3 Table), respectively.”

5. Table 6 compared the predictive performance of the conventional and new models.

5.1. List the factors included in both models in the text of the footnote of the table.

**→ Response:** The factors including in both models were described in the text of the footnote of the table. “Conventional model includes gestational age (weeks), pre-pregnancy body mass index (BMI) (kg/m^2^), parity (the number of deliveries), newborn’s gender and gestational diabetes (yes/no); new model includes binge drinking before pregnancy plus conventional model.” (**Page. 19, Table 6, footnote, line 350-352**)

5.2. Describe how the cutoffs (low, mid-low, mid-high, high) were chosen.

**→ Response:** As pointed out, the NRI depends on the specific risk category used, especially, it may be influenced by the number and choice of cut-off for identifying the risk category. So, the best choice of risk categories for reclassification seems to be an important. Category cut-offs were based on deciles of the distribution of absolute risk for macrosomia. NRI statistics were evaluated by cut-offs with varying numbers of intervals for four risk categories. The NRI statistics in various numbers of the intervals (2~5%) and various cut-off points of high risk (from 10 to 15% by 1%) were tested. These results showed that good prediction of incident macrosomia with the new model rather than the conventional model was robust to various cut-off points tested**.(Page 19, Table 6 footnotes, line 347-350)**

**<Discussion>**

1. In the second paragraph of this section, I recommend discussing the lab results in table 2 when discussing the association between alcohol drinking before pregnancy and maternal hemostasis.

**→ Response**: According to your suggestions, we have discussed about lab-clinical data in table 2 and described in **page 22, line 406-411**. “Lab-clinical data also show that women with binge drinking pattern before pregnancy exhibited significant increases of third-trimester fasting glucose, not in first-trimester, compared to non-binge drinking groups. The elevation of fasting glucose in binge drinking groups of the third trimester was correlated with significant increases of hemoglobin, hematocrit, ALT, total protein, and albumin levels compared to non-binge drinking groups. Our data provides solid evidence of an independent association between macrosomia and alcohol drinking before pregnancy regardless of the influences of traditional risk factors that can affect the development of macrosomia.”

2. I recommend discussing whether the authors evaluated binge eating/eating disorders among those participants. Binge eating/eating disorders before and during pregnancy might be associated with both binge drinking and macrosomia, which might impact the results of the association between binge drinking and macrosomia.

**→ Response**: According to your suggestion, we have discussed the relationship between binge eating and binge drinking before or during pregnancy and its-mediated macrosomia. **Described in Page 23-24, line 431-443.** In the present study, we didn’t consider the relationship between binge eating (or eating disorders) and binge drinking (and its-medaited macrosomia). However, it could be important to examine the relationship between eating patterns before pregnancy and binge drinking behavior, resulting in an increased risk of macroaomia. Therefore, it may be further needed to establish the data by requesting the additive survey examination and evaluation about eating disorders (binge eating) in Korean pregnancy registry cohort for future study.

**Description as follows:** “On the other hand, a recent study demonstrated that binge eating before or during pregnancy is associated with prematurity, macrosomia, and future risk of diabetes and metabolic syndrome in infants as well as with higher gestational weight gain and greater postpartum weight retention in mother [53]. In addition, some studies demonstrated that it is not only the type of diet (i.e., frequency of fat intake) but also the type of eating behavior (i.e., binge eating) that seems to contribute to explaining binge drinking [54]. Along the same line, binge eating behaviors may be associated with binge drinking and could be a gateway to the initiation and escalation of binge drinking, resulting in an increased risk of macrosomia. Nevertheless, studies on pregnancy and neonatal outcomes among women with ongoing or previous eating disorders are scare. Unfortunately, our present study also didn’t consider the relationship between binge eating (or eating disorders) and binge drinking (and its-mediated macrosomia). However, it could be important to examine the relationship between eating patterns before pregnancy and binge drinking behavior, resulting in an increased risk of macroaomia. Therefore, it may be further needed to establish the data by requesting the additive survey examination and evaluation about eating disorders (binge eating) in Korean pregnancy registry cohort for future study.”

II. Reviewer #2 :

**<Introduction>**

1. This section references reports from the CDC and other studies that appear to be focused on US women, however there are also comments on alcohol consumption in different countries. Recommend that authors are clear what country/population they are citing as it is important to report the statistics with that additional context.

**→ Response:** As you suggested, the prevalence of alcohol consumption during pregnancy in each country was added and described in **Page 3, line 42-50.**

Description is as like: “Although reported rates of alcohol use among young women vary depending on the differences in cultural factors each country, more than 50% of young women in many countries drink alcohol before pregnancy [3, 4]. Despite the recommendations of alcohol abstinence for women who are pregnant or planning a pregnancy [5, 6], previous systematic review and meta-analysis studies demonstrated that alcohol use during pregnancy is common in many countries; especially, Russia (36.5%, the United Kingdom (41.3%), Denmark (45.8%), Belarus (46.6%) and Ireland (60.4%) showed the highest rates [7]. In the United States, approximately 10% of pregnant women admit to alcohol drinking in the past month, and about 50% of them still admit to drinking at some point during their first trimester, often prior to being aware that they are pregnant [8], and in Australia, where pregnant women are recommended not to drink alcohol at any point during pregnancy, 72% did so [9].”

2. Authors commented that the impact of ethanol consumption pre-conception on fetal development and postnatal growth is unclear. Recommend that it is important to highlight that the first trimester of pregnancy is the most susceptible to teratogens and that a large % of women are unaware they are pregnant until 4-6 weeks gestation.

**→ Response:** As you suggested, we described the impact of alcohol consumption before pregnancy or during early pregnancy for the altered fetal development and growth at an early period of pregnancy. And also, we described the percentage of women who drink alcohol in the preconception period do not recognize pregnancy until the fourth to sixth week of gestation. Describe in **Page 3-4, line 51-62**. Description is as like: “Consequently, alcohol drinking before pregnancy may be closely associated with unintended fetal alcohol exposure, and it may be a causal factor for detrimental maternal and fetal health. Several studies demonstrated that alcohol is teratogenic and fetotoxic, and passes freely across the placenta to the unborn baby at levels at least equal to that of the mother [13]. It is also widely acknowledged that prenatal alcohol exposure can have a negative impact on growth before and after birth, miscarriage, stillbirth, and preterm birth [14, 15]. However, up to 60% of women who drink alcohol in the preconception period do not recognize pregnancy until the fourth to sixth week of gestation [16]. In fact, it is well known that early stage of pregnancy is an important period to prepare for homeostasis of energy metabolism required for fetal development or growth [17]. In particular, since most major organs, such as the limbs, eyes, and ears, start to form and develop at an early stage [158], acute or chronic alcohol consumption before pregnancy or during early pregnancy may alter the first adjustment of fetal development and growth and may trigger the teratogenic effects of alcohol consumption-mediated fetal developmental disorders.”

<**Methods>**

3. It is important to clarify that diseases excluded are "pre-gestational" diabetes mellitus - so to not confuse with gestational diabetes mellitus.

**→ Response:** According to your comment, we changed from diabetes mellitus to **pre-gestational diabetes mellitus** (**Page 6, line 107**).

4. Unclear why only hepatitis A and B are excluded, but not hepatitis C or other underlying causes of liver-failure unrelated to alcohol consumption

**→ Response:** In our pregnancy registry, women with hepatitis C were not found, unlike those with hepatitis A or B, so we did not include them in exclusion list to determine the study target group.

5. Smoking is listed as a "traditional risk factor" for macrosomia, however the literature on smoking suggests it is associated with fetal growth restriction and preterm birth. If smoking is highlighted, then polysubstance use would be appropriate to note for all the participants too.

**→ Response**: Your point is correct. As like in your suggestion, several previous studies demonstrated that maternal smoking was associated with decreased risk of macrosomia and also it was associated with increased risk of low birth weight. Smoking and physical activity are modifiable lifestyle risk factors and thus they are not included in traditional risk factors of macrosomia. Therefore, the sentence are changed and corrected as described in **page 7-8, line 137-139** as follow: “Traditional risk factors were classified according to following as: Maternal age (<35, low-risk; ≥35, high-risk), Prepregnancy BMI (<25, low-risk; ≥25, high-risk), Parity (nulliparous, low-risk; ≥1, high-risk), GDM (No, low-risk; Yes, high-risk). ^§^There is no subjects with GDM, who has binge drinking status (n=0).”

And also, description for **separated smoking and physical activity** are shown in **Page 8, line 148-151** as follows: “In addition, modifiable lifestyle factors were classified according to following as: Smoking before and during pregnancy (Former or current, low-risk; None, high-risk), Physical activity before and during pregnancy (More than moderate, low-risk; None or light, high-risk); and analyzed their effects on the risk of developing macrosomia by maternal alcohol drinking status.”

6. Need to define the acronym GDM - gestational diabetes - before using it and also clarify how this was diagnosed (eg. 1hr or 2hr glucose tolerance test, HbA1c?)

**→ Response:** As you suggested, the diagnostic criteria for gestational diabetes are presented as follows **(described in page 8, line 140-148)**. “GDM was diagnosed using a two-step method as described in previous study [40]. Briefly, universal screening with a 50-g glucose challenge test (GCT) was conducted between 24 and 28 weeks. If the result of the GCT was 140 mg/dL or more, a confirmatory oral glucose tolerance test (OGTT) was performed. The Cheil General Hospital used the 75-g OGTT as the confirmatory test and used the new diagnostic criteria from the International Association of Diabetes and Pregnancy Study Group (at least on abnormal value: fasting glucose ≥92 mg/dL, 1-hour glucose ≥180 mg/dL, or 2-hour glucose ≥153 mg/dL). The CHA Gangnam Medical Center used the 100-g OGTT and the Carpenter-Coustan criteria for GDM diagnosis (2 or more abnormal value: fasting glucose ≥95 mg/dL, 1-hour glucose ≥180 mg/dL, 2-hour glucose ≥155 mg/dL, or 3-hour glucose ≥140 mg/dL). The participants who were diagnosed GDM were sent to endocrinologist and management methods were determined after clinical evaluation.” Unfortunately, this registry cohort did not performed the examination of HbA1c and it was not included in lab test item.

7. Need to explain how congenital anomalies were detected, is this based on in-utero ultrasound or routine fetal anatomic survey, or postnatal assessment

**→ Response**: As you suggested, the diagnosed method of congenital anomalies was described in **Page 8, line 151-162**. Description as follow: “Also, congenital anomalies are diagnosed before and after birth through imaging methods, such as ultrasound or fetal magnetic resonance imaging (MRI) [41]. Ultrasound examinations were performed at approximately 20 weeks of gestational age. An additional level II ultrasound examination or fetal magnetic resonance imaging (MRI) was performed if fetal anomalies were found on ultrasound examination. Especially, before the fetal MRI was performed, all participants were informed in writing or orally about the safety of the technique and the process and method of the procedure, and the fetal MRI was performed only when they understood and gave their consent. The radiologist was provided with information on the clinical history and the findings of the detailed ultrasound. The results of the ultrasound and fetal MRI were discussed by the specialized radiologist, neonatologists, and obstetricians of the Cheil and CHA hospitals. Complementary invasive tests such as aminocentesis and chronic villus sampling (CVS), which are highly sensitive and specific for the diagnosis of chromosomal or genetic disorders of the fetus and infection risk, are also performed for accurate diagnosis [41]. As well, maternal blood can be used to screen for placental markers to aid in prediction of risk of chromosomal abnormalities or genetic defects [41].”

**<Results>**

8. Page 12, Instead of referencing number of drinks = "cups" - recommend authors use a volume (e.g. drinks - 8oz)

**→ Response:** As you suggested, the number of drinks (cup) may re-calculated into the volumes or weight (ounce, oz). Ounce is a unit of weight equal to approximately 28.349 grams. We considered that a standard drink contains 12 grams of pure alcohol in any drink (described in page 7, line 118). Based on this, the 10, 20, and 30 drinks/cups were calculated as 4.2 oz, 8.5 oz, and 12.7 oz, respectively. In the progression of surveillance for the establishment of Korean pregnancy registry, the amount of alcohol drinking was investigated in cup unit, so drinks was expressed in both cups and ounces. These are described in **Page 7, line 117-123 (methods section)** as follows: “In these questions, a ‘drinks’ means the standard cup for each type of drink. In this study, we considered that a standard drink contains 12 grams of pure alcohol in any drink [36]. Among ever drinker (former or current), the number of drinks (cup) consumed per month was calculated by using frequency of alcohol-drinking and quantity of alcohol-drinking per drinking day before pregnancy. And also, the number of drinks (cups) was re-calculated into the ounce (oz), a unit of weight equal to approximately 28.349 grams. Firstly, the participants were categorized according to the number of drinks (cup or ounce) consumed per month as never drinking, ≤10 cup (4.2 oz), 10 cup (4.2 oz)< - ≤20 cup (8.5 oz), 20 cup (8.5 oz)< - ≤ 30 cup (12.7 oz), and >30 cup (12.7 oz).”

And also, the text in Results section (**Page 15, line 267-271**) and the legend of Fig 1B (**Page 16, line 284-287)** were changed.

**<Discussion>**

9. Authors reference a former rat study regarding fetal macosomia/metabolics, however there has been a recent non-human primate study of alcohol consumption (Lo et al. AJOG 2021) that noted no difference in fetal weight between controls and alcohol-exposed. Recommend citing other more recent and translational animal studies.

**→ Response:** As you suggested, we cited more recent studies for the relationship between alcohol consumption and birthweight. Describe in **page 22, line 389-391** and add in references **47**.

**<Tables>**

10. In Table 1, it would be interesting to also include maternal race/ethnicity as well as other polysubstance use besides smoking

→ **Response**: I fully agree with your suggestion to include the mother's race/ethnicity. However, all of participants included in our pregnancy cohort consisted only of Koreans, so we did not include them because there was no significant difference in nationality or race.

11. Recommend using the words "vaginal delivery" rather than "normal delivery"

→ **Response**: In **Table 3**, we have changed from “Normal delivery” into “vaginal delivery”**(Page 14)**

12. In Table 2: Would be more relevant to include maternal HbA1c values rather than a random glucose as part of the comprehensive metabolic panel

→ Response: Absolutely, I agree with your comments. Unfortunately, this registry cohort did not performed the examination of HbA1c and it was not included in lab test item. Thereafter, we have been newly established another pregnancy cohort with more expanded participants and examination items from 2020. Once this new cohort is enrolled, a broader range of investigative analyzes could produce clearer evidence related to maternal and fetal health.

13. In Table 3: Need to define criteria for gestational diabetes as well as pregnancy-induced hypertension in the text or table legend

**→ Response**: As you suggested, the definition for GDM and pregnancy-induced hypertension are described in the Method (text)(for GDM**) (page 8, line 140-148)** and table 3 legend (for pregnancy-induced hypertension**)(page 14, line 246-247)**.

14. In Table 4:

14.1. Recommend adding 1 and 5 min apgar scores to this table

**→ Response:** As you suggested, we have added apgar score (1 min and 5 min) (Table 4)(page 16). However, there were no difference in apgar scores for 1 and 5 minutes between offspring from women with binge drinking and never or non-binge status (described **page 15, line 260-262**). Description is as like: “However, there were no difference in gender, height, head circumference, glucose, the prevalence of congenital anomaly, and apgar scores for 1 and 5 minutes between offspring from women with binge drinking and never or non-binge status.”

14.2. Recommend clarifying the admission is to the "neonatal intensive care unit"

→ Response: As you suggested, we have changed into “Admission to **neonatal** intensive care unit”. (in **Text, Page 15, line 259** and **Table 4, Page 16**)

14.3. Instead of writing "boys" or "girls" recommend using "male" and "female"

→ Response: As you suggested, we have changed from “boys and girls” into **“male and female”**. **(Page 16, Table 4)**
