# Supplementary material for: Binge alcohol drinking before pregnancy is closely associated with the development of macrosomia: Korean pregnancy registry cohort
Source: PLoS One. 2022 Jul 12;17(7):e0271291. doi: 10.1371/journal.pone.0271291 (PMC9275693; doi:10.1371/journal.pone.0271291)
Supplement: Attachment — Submitted filename: Comments for the revision.docx [file pone.0271291.s009.docx]

The authors had improved their manuscript. I have two further questions regarding their responses.

4. In the following sentence: “If women answered ‘Former drinker’ or ‘Current drinker’, they were then further

asked average frequency of alcohol-drinking (≤1/month, 1-2/month, 2-3/week, ≥4/week, everyday) and

quantity of alcohol-drinking per drinking day (1-2 drinks, 3-4 drinks, 5-6 drinks, 7-9 drinks, ≥10 drinks).”

Were pregnant women asked about their average frequency of alcohol consumption in their lifetime? in the

past year? 6 months? Or 1 month?

→ **Response:** In cohort, we collected the information on maternal alcohol consumption before and during

pregnancy through a questionnaire for lifetime drinking, past year, 6 months, recent 1month, current drinking,

duration, and amount at the first antenatal care visit and also continuously evaluated at each visit. In this study,

the average frequency of alcohol drinking and quantity of alcohol-drinking per drinking day were evaluated

based on the result for a specific questionnaire in their lifetime before pregnancy. In fact, there was few

participant to drink alcohol during the pregnancy (n=3) in this cohort. **(Described in page 7, line 117)**

**Comments: Why was the lifetime alcohol drinking pattern instead of the past year or 6 months drinking pattern selected? The past year or 6 months drinking pattern might be more relevant to pregnancy. I am asking because alcohol drinking patterns can vary a lot in different age groups (e.g., 20s vs. 30s).**

5. In the “definition of macrosomia and its risk factors” section, I recommend specifying whether “smoking” and “physical activity” refer to smoking and physical activity before pregnancy or during pregnancy. Provide definitions of different levels of physical activity.

→ **Response:** As like in evaluation of alcohol drinking, smoking and physical activity were also assessed at each visit. Smoking was queried by the following items such as lifetime, last month, and current smoking and their duration and amount. And also, physical activity was queried at each visit for physical activity before and during pregnancy (frequency and duration of walking, moderate and vigorous-intensity activity). So, the results for smoking or physical activity in their lifetimes including before and during pregnancy were used for assessing infant outcome, macrosomia.

Since smoking and physical activity are not included in traditional risk factors of macorosomia, we described in **page 8, line 148-151** as follows: “In addition, modifiable lifestyle factors were classified according to following as: Smoking before and during pregnancy (Former or current, low-risk; None, high-risk), Physical activity before and during pregnancy (More than moderate, low-risk; None or light, high-risk); and analyzed their effects on the risk of developing macrosomia by maternal alcohol drinking status.”.

**Comments: I might have misunderstood. But why** “**Former or current” smokers were low-risk and never smokers were high-risk?. Also, I saw how you categorized different levels of physical activity in the footnote of Table 1, but not in the main text. This should be added to the main text as well.**
