# Supplementary material for: Binge alcohol drinking before pregnancy is closely associated with the development of macrosomia: Korean pregnancy registry cohort
Source: PLoS One. 2022 Jul 12;17(7):e0271291. doi: 10.1371/journal.pone.0271291 (PMC9275693; doi:10.1371/journal.pone.0271291)
Supplement: Attachment — Submitted filename: Response to reviewer (2nd revision).docx [file pone.0271291.s010.docx]

June 9, 2022

Editor Board

*PLOS ONE*

Dear Editors,

I would like to re-submit a revised version of the manuscript for publication in *PLOS One*, titled ‘Binge alcohol drinking before pregnancy is closely associated with the development of macrosomia: Korean pregnancy registry cohort’. The manuscript ID is PONE-D-21-31508R1.

We appreciate you giving us the opportunity to revise our manuscript. We have carefully considered each of the editor’s and reviewers’ comments and suggestions, which helped us improve our manuscript. We have provided point-by-point responses to all the reviewers’ comments in the attached letter.

We hope that our revised manuscript is now suitable for publication in *PLOS One*. We look forward to hearing from you.

Sincerely yours,

Won-Ho Kim, Ph.D.

Director

Division of Cardiovascular Disease Research,

Department of Chronic Disease Convergence Research, National Institute of Health,

187, Osong Saengmyeong 2-ro, Osong-eup, Cheongju city,

Chungbuk, Korea, 28159,

Tel: +82-43-719-8650; Fax: +82-43-719-8689; E-mail: [jhkwh@nih.go.kr](mailto:jhkwh@nih.go.kr)

**Responses to the Editor’s and Reviewers’ Comments**

I. **EDITOR’S COMMENTS TO AUTHOR:**
1. Please include the following items when submitting your revised manuscript:
   1.1. A rebuttal letter that responds to each point raised by the academic editor and reviewer(s). You should upload this letter as a separate file labeled 'Response to Reviewers'.

**→ Yes, we have uploaded with a separate file labeled “Response to Reviewers”.**

2.2. A marked-up copy of your manuscript that highlights changes made to the original version. You should upload this as a separate file labeled 'Revised Manuscript with Track Changes'.

**→ Yes, we have uploaded with a separate file labeled “Revised Manuscript with Track Changes”.**

1.2. An unmarked version of your revised paper without tracked changes. You should upload this as a separate file labeled 'Manuscript'.

**→ Yes, we have uploaded with a separate file labeled “Manuscript”.**

2. **We have carefully checked the typographical or grammatical errors and corrected in the text. Described with red color.**

**II. Reviewer #1 :**

**<In Method>**

1. Why was the lifetime alcohol drinking pattern instead of the past year or 6 months drinking pattern selected? The past year or 6 months drinking pattern might be more relevant to pregnancy. I am asking because alcohol drinking patterns can vary a lot in different age groups (e.g., 20s vs. 30s).

**→ Response:** We absolutely agree with your comments that using a drinking pattern of the past year or 6 months instead of the lifetime drinking pattern may be more effective in determining fertility or effects on the development of fetus during the pregnancy or the growth of infants. In cohort, we collected the information on maternal alcohol consumption before and during pregnancy through a questionnaire for lifetime drinking, past year, 6 months, recent 1month, current drinking, duration, and amount at the first antenatal care visit and also continuously evaluated at each visit. Our cohort’s information on maternal alcohol consumption were collected via maternal self-report according to each specific questionnaire or interview-based questionnaires, which could have missing data and led to potential bias. Particularly with respect to smoking and alcohol consumption before or during pregnancy, self-reports of substance use may have underestimated actual use due to the negative perception and stigmatization. In fact, the questionnaire on alcohol drinking included lifetime drinking and past 1 year and 6 months, current drinking, duration, and amount; however, data on the frequency and amount of drinking for the past 1 year and 6 months were not accurately collected due to related data missing. So, our study used survey and analysis data for lifetime drinking instead of those for past 1 year and 6 months. Meanwhile, another pregnancy cohort that is currently being constructed has more accurate data than the existing cohort, future studies using these data will be able to provide more accurate and specific results than now.

**Described in discussion (page 25, line 482-**491).

And also, I fully agree with the reviewer's comment that drinking pattern can vary significantly with age (20s vs. 30s). Therefore, the drinking patterns according to age were analyzed and presented in Table 1 (page 11). Our pregnancy cohort consisted of 16.9% of women in their 20s, 46.8% of 30-34 years-old, 30.6% of 35-39 years-old, and 5.7% of those over 40 years-old. Firstly, binge drinking pattern in the 20s was 9.2%,while the 30-34 years and 35-39 years-old showed a significant decrease to 8.1% and 6.7%, respectively (p<0.042). However, in the non-binge drinking group, there was no significant difference in the distribution between those in their 20s and 30s. The proportion of never drinkers showed a slight increase in the 30s compared to the 20s (below figure, but not presented in main text.). The sample size classified by age was too small for statistical analysis.

0

20

40

60

80

100

Drinking pattern distribution (%)

Never drinking

(n=561)

Non-binge drinking

(n=2099)

Binge drinking

(n=226)

≤29

30-34

35-39

≥40

P<0.045

P<0.042

P=0.743

2. I might have misunderstood. But why “Former or current” smokers were low-risk and never smokers were high-risk?.

**→ Response:** The reason for using former or current-smokers as a high-risk group instead of never-smokers is that previous several papers had been reported that smoking during pregnancy is closely associated with infant weight loss and even reduces the incidence of macrosomia. Although recent some studies found no crude or adjusted association between maternal smoking and macrosomia (58-60), most of early studies reported that maternal smoking was associated with decreased risk of macrosomia (41-43). However, in Table S4, there was no difference in the risk of developing macrosomia between the high-risk non-smoking group and the low-risk group for former or current smoking. On the other hand, when both groups for former or current smoking and non-smoking were exposed to binge drinking before pregnancy, the risk of macrosomia was significantly increased into 6.8 and 7.9 folds, respectively (Fig. S2A).

**The relationship about between smoking and macrosomia was described in Discussion** (Page 24, line 449-457). And also, the references explain the relationship between maternal smoking and macrosomia were added in line 150 (41-43), 452(41-43), 453(58-60) in the text and in reference section.

3. Also, I saw how you categorized different levels of physical activity in the footnote of Table 1, but not in the main text. This should be added to the main text as well.

**→ Response:** The categorization for different levels of physical activity will be also added in the method column of the main text. “Physical activity before and during pregnancy was queried at the first antenated visit and further asked at each visit for frequency and duration of walking and moderate- and vigorous-intensity activity. Based on these questionnaires, physical activity levels were categorized according to the following criteria: None or light in physical activity (almost sedentary lifestyle, office work, and a housewife with few housework, etc.) and More than moderate in physical activity (manufacturing, architect, farmer, athlete, housewife with lots of housework, etc.).” (**Described in Page 8, line 151-156.)**

II. Reviewer #2 :

**<Introduction>**

1. As there are no women with hepatitis C in the pregnancy registry, it is more comprehensive to say that patients with Hepatitis A, B and C were excluded from the study. Rather than just Hepatitis A and B alone.

**→ Response:** As you suggested, in addition to those with hepatitis A and B, those with hepatitis C was also excluded from the enrollment of study subjects. It was described in Study participants section of Methods as follows: “hepatitis A, hepatitis B, or hepatitis C” (Described in **Page 6, line 109).**

2. Even though the patient population was all Korean and that's why race/ethnicity was not reported, it is important to state this in the results section as part of the demographics. Otherwise, it will appear like the study did not further analyze by race.

**→ Response:** **:** As you pointed out, the following reasons were presented for not identifying racial differences. **Described in page 12, line 218-219 as follows**: “On the other hand, since all subjects participated in this study were Korean, the racial comparison results could not presented.” As well, we also described in discussion as follows: Described in Page 26, line 496-497 “As well, since all subjects participated in this study are Korean, no interracial comparison results were presented.”

3. Page 18, line 321: correct (S2 Table → S4 Table)
4. The numbering for Reference was changed in the text and reference. (red color)
